# Incongruence between confirmed and suspected clinical cases of Japanese encephalitis virus infection

**DOI:** 10.3389/fcimb.2024.1302314

**Published:** 2024-01-26

**Authors:** Wei Li, Yuliang Feng, Hongrong Zhong, Mingfeng Jiang, Jiake Zhang, Shihua Lin, Na Chen, Shusen He, Kai Zhang, Shihong Fu, Huanyu Wang, Guodong Liang

**Affiliations:** ^1^ Institute of Microbiological Detection and Analyses, Sichuan Center for Disease Control and Prevention, Chengdu, China; ^2^ Institute of Immunization Programme, Sichuan Center for Disease Control and Prevention, Chengdu, China; ^3^ Department of Arbovirus, National Key Laboratory of Intelligent Tracking and Forecasting for Infectious Diseases, National Institute for Viral Disease Control and Prevention, Chinese Center for Disease Control and Prevention, Beijing, China

**Keywords:** Japanese encephalitis, Japanese encephalitis virus, viral encephalitis, enterovirus, Epstein-Barr virus, unknown viral encephalitis, viral encephalitis in adults

## Abstract

**Background:**

Japanese encephalitis (JE) is a notifiable infectious disease in China. Information on every case of JE is reported to the superior health administration department. However, reported cases include both laboratory-confirmed and clinically diagnosed cases. This study aimed to differentiate between clinical and laboratory-confirmed cases of Japanese encephalitis virus (JEV) infection, and improve the accuracy of reported JE cases by analyzing the acute-phase serum and cerebrospinal fluid of all reported JE cases in the Sichuan province from 2012 to 2022.

**Methods:**

All acute-phase serum and/or cerebrospinal fluid samples of the reported JE cases were screened for IgM(ImmunoglobulinM)to JEV using the enzyme-linked immunosorbent assay (ELISA), and the detection of the viral genes of JEV and 9 other pathogens including enterovirus (EV), using reverse transcription PCR was attempted. Epidemiological analyses of JE and non-JE cases based on sex, age, onset time, and geographical distribution were also performed.

**Results:**

From 2012 to 2022, 1558 JE cases were reported in the Sichuan province. The results of serological (JEV-specific IgM) and genetic testing for JEV showed that 81% (1262/1558) of the reported cases were confirmed as JEV infection cases (laboratory-confirmed cases). Among the 296 cases of non-JEV infection, 6 viruses were detected in the cerebrospinal fluid in 62 cases, including EV and the Epstein-Barr virus (EBV), constituting 21% (62/296) of all non-JE cases. Among the 62 non-JEV infection cases with confirmed pathogens, infections with EV and EBV included 17 cases each, herpes simplex virus (HSV-1/2) included 14 cases, varicella- zoster virus included 6 cases, mumps virus included 2 cases, and human herpes viruses-6 included 1 case. Additionally, there were five cases involving mixed infections (two cases of EV/EBV, one case of HSV-1/HSV-2, one case of EBV/HSV-1, and one case of EV/herpes viruses-6). The remaining 234 cases were classified as unknown viral encephalitis cases. Our analysis indicated that those aged 0–15 y were the majority of the patients among the 1558 reported JE cases. However, the incidence of laboratory-confirmed JE cases in the >40 y age group has increased in recent years. The temporal distribution of laboratory-confirmed cases of JE revealed that the majority of cases occurred from May to September each year, with the highest incidence in August.

**Conclusion:**

The results of this study indicate that there is a certain discrepancy between clinically diagnosed and laboratory-confirmed cases of JE. Each reported case should be based on laboratory detection results, which is of great importance in improving the accuracy of case diagnosis and reducing misreporting. Our results are not only important for addressing JE endemic to the Sichuan province, but also provide a valuable reference for the laboratory detection of various notifiable infectious diseases in China and other regions outside China.

## 1 Introduction

Japanese encephalitis (JE) is a viral disease transmitted by mosquitoes that can lead to severe encephalitis. JE involves fever, meningitis, encephalitis, and meningoencephalitis (Pierson and Diamond, 2013). Japanese encephalitis virus (JEV) is carried by various mosquito species, but *Culex tritaeniorhynchus* is its primary vector. In nature, birds and pigs act as intermediate and reservoir hosts of JEV, respectively, while humans are the final hosts (Solomon, 2006; Pierson and Diamond, 2013; Griffiths et al., 2014). The fatality rate among JE patients is approximately 20–30%, while 30–50% of survivors may endure neurological sequelae, such as severe consciousness disturbance, dementia, aphasia, and limb paralysis (Solomon, 2006; Griffiths et al., 2014). According to the statistical data provided by the World Health Organization, JE is prevalent in 24 countries and territories in Asia and Oceania; approximately 3 billion people live in JE-endemic areas and are at risk of JEV infection. Nearly 67,900 JE cases and 10,000 deaths occur annually (Campbell et al., 2011). JE can be prevented through vaccination; with the implementation of JE vaccination programs in children in JE endemic areas, both Japan and Korea have reduced the number of JE cases to almost single digits (Pierson and Diamond, 2013; Halstead and Jacobson, 2018) However, due to increased global interactions, population movement, and increased transportation of goods, the geographic range of JEV has expanded, leading to local infections in Africa (Simon-Loriere et al., 2017) and the incidence of JEV-positive birds and mosquitoes in Europe (Platonov et al., 2012; Ravanini et al., 2012). JE has become a mosquito-borne infectious disease that is a public health issue of global concern.

JE is highly endemic in China (Zheng et al., 2012; Chen et al., 2021). Since 1950, JE has been included in the national notifiable infectious disease list in China; since then, information on every case of JE has been reported to the superior health administration department. According to statistics, from 1950 to 2018, a total of 2,364,177 JE cases with 275,792 deaths were reported in China, the average number of cases reported annually was 34,263, and the average number of deaths annually was 3,997 (Chen et al., 2021). China successfully developed JE-inactivated (P3) and JE-attenuated live (SA-14-14-2 strain) vaccines in the 1960s and the 1980s, respectively, which have been gradually applied nationwide. Thereafter, the incidence of JE has reduced substantially (Gao et al., 2014). Since the Chinese government included the JE vaccine in the Expanded Program on Immunization in 2008, a significant decrease in reported JE cases has been observed; in 2021 and 2022, there were 209 and 153 reported JE cases nationwide, respectively (Division of Infectious Disease, Chinese Center for Disease Control and Prevention, 2022). It can be seen that the public health burden of JE in China is decreasing yearly.

The Sichuan province is located in southwest China, between 97°21’E and 108°33’E (spanning 11°12’) and 26°03’N and 34°19’N (spanning 8°16’). It has a warm, humid climate with abundant rainfall, providing an excellent natural environment for the breeding of mosquitos. Moreover, rice production is prevalent in the Sichuan province, making these areas favorable for mosquito breeding, especially *C. tritaeniorhynchus*, which is the main vector of JEV. *C. tritaeniorhynchus* is also a dominant species in the Sichuan province (Hu et al., 2017; Li G. et al., 2019). Additionally, the Sichuan province leads in pig production in China, creating favorable conditions for the amplification of JEV in the natural environment. Owing to these factors, JE has been highly endemic in the Sichuan province (Zheng et al., 2012; Chen et al., 2021). After the Sichuan province included the JE vaccine in the Expanded Program on Immunization in 2008, there has been a marked decrease in JE incidence owing to extensive vaccination. However, from 2002 to 2010, JE remained highly endemic in the Sichuan province, with an average annual incidence rate of 1/100,000 (Wang et al., 2013). To strengthen the prevention and control of JE in the Sichuan province, a JE surveillance network system covering all 21 cities (prefectures) was established toward the end of 2011 (hereafter referred to as the network laboratory). The network laboratory conducts testing training for local primary laboratories annually and encourages them to confirm every reported JE case in their respective cities (prefectures). Since 2012, the laboratory confirmation rate of JE cases reported to the superior health administration department in Sichuan province has increased annually. However, the Sichuan province is a predominantly mountainous area with imbalanced economic development, and there are inconsistencies in the laboratory detection conditions and capabilities among the 21 cities (prefectures). As a result, the reported JE cases included cases clinically diagnosed in local primary laboratories, and not all of them were confirmed through laboratory testing. To study the gaps between clinical and confirmed cases of JEV infection, and improve the accuracy of reported JE cases, we conducted serological (JEV-specific IgM) and genetic analysis of the acute-phase serum and cerebrospinal fluid (CSF) of all reported JE cases in the Sichuan province from 2012 to 2022.

## 2 Materials and methods

### 2.1 Specimen collection and preservation

**Figure 1:**
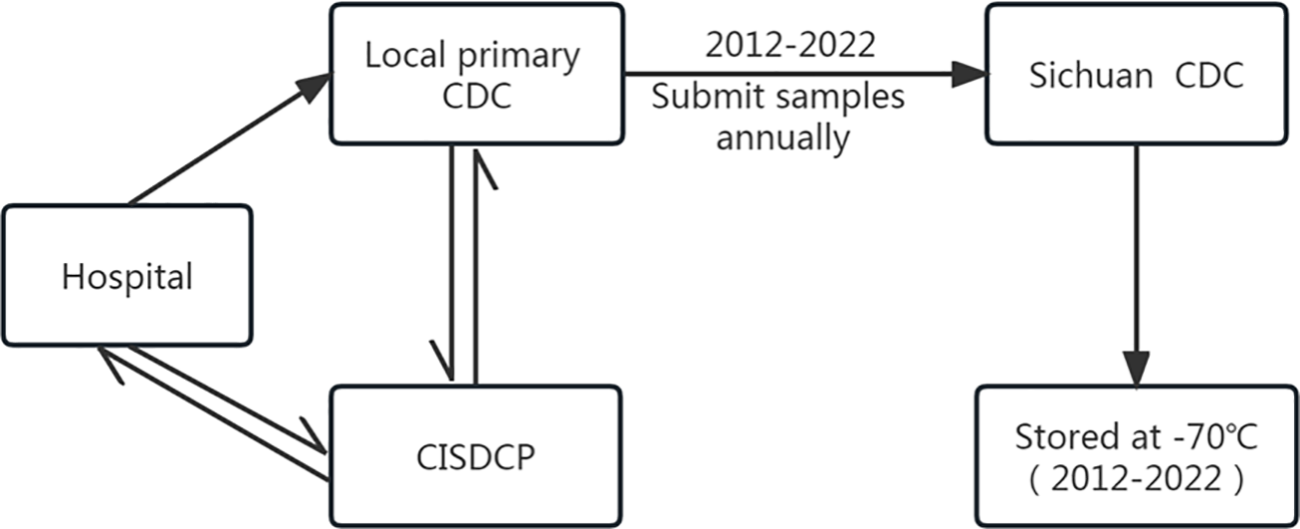
Flowchart of specimen collection and delivery in the JE surveillance network system in the Sichuan province of China.

By the end of 2011, the Sichuan province had established a comprehensive JE surveillance network that covered hospitals at all levels and the centers for disease control and prevention (CDC). According to this system, when JE cases (including suspected cases) are reported to the China Information System for Disease Control and Prevention (CISDCP) by hospitals, the local primary CDC is responsible for conducting epidemiological investigations, collecting acute-phase specimens (specimens collected within 7 d of the onset of illness, including serum and CSF) from patients, and performing laboratory testing for JEV quickly when they notice the information in the CISDCP. The results of JEV testing are uploaded to the CISDCP by the local primary CDC, while the doctors in hospitals are notified of the results immediately through the CISDCP. The system also requires each local primary CDC to send the collected patient specimens to the Sichuan CDC. All specimens used in this study were submitted by the local primary CDC within the JE surveillance network in the Sichuan province. Specimens were stored at -70 °C in the freezer at the Sichuan CDC (**Figure 1**).

### 2.2 Data collection and management

The JE data used in this study were obtained from CISDCP. These data include the number of reported cases, annual incidence, and mortality, as well as demographic information of JE cases, such as age and sex, in the Sichuan province from 2012 to 2022.

### 2.3 Detection of JEV IgM using enzyme-linked immunosorbent assay

All JE cases were confirmed following the “Diagnostic Criteria for Japanese encephalitis, WS214-2008” by the Ministry of Health of the People’s Republic of China (National Health Commission of the People’s Republic of China, 2008). All acute-phase serum and/or CSF samples from reported JE cases were screened for IgM against JEV using the JEV IgM-capture ELISA kit (Shanghai B& C Enterprise Development Co. Ltd, Shanghai, China) (Wang et al., 2007).

### 2.4 Nucleic acid extraction and PCR

Reverse transcription PCR (RT-PCR) was performed to detect JEV genes in the CSF of the patients. Additionally, according to the “expert consensus on the application of pathogenic diagnostic techniques for viral encephalitis and meningitis” provided by the China Primary Health Care Foundation Professional Committee of Pathogen Detection in 2023 (Xu et al., 2023), JEV-negative CSF samples were further analyzed to detect other viruses such as the enterovirus (EV), varicella- zoster virus (VZV), Epstein-Barr virus (EBV), mumps virus (MuV), human herpes viruses-6 (HHV-6), herpes simplex virus (HSV), measles virus (MV), rubella virus (RUV), and cytomegalovirus (CMV). Total RNA was extracted from the CSF using the Maxwell^®^16 viral RNA extraction kit (Promega, Madison, Wisconsin, USA) according to the manufacturer’s instructions. RT-PCR for JEV and nine other viruses was performed using a real-time PCR detection kit (Beijing Zhuo Cheng Hui Sheng Biotechnology Co., Ltd., Beijing, China) (Liu et al., 2022).

### 2.5 Analysis of epidemiological characteristics

The epidemiological characteristics in different age groups among the reported cases, laboratory-confirmed JE cases, non-JE cases with confirmed pathogens, and unknown viral encephalitis cases (four groups of viral encephalitis) from 2012 to 2022 were presented in the form of line and bar charts to illustrate the trends, including the annual incidence and mortality, monthly incidence, number of cumulative cases, sex ratio, season patterns, etc. To observe differences in the three groups of viral encephalitis epidemiological features in different age groups, the cases were grouped into three categories, namely, 0–15, 16–40, and >40 y of age.

We used a series of thematic maps, based on JE incidence data from the Sichuan Province, between 2012 and 2022, to analyze the spatial and temporal patterns of reported JE cases and the geographical distribution of non-JE cases with confirmed pathogens and unknown viral encephalitis cases.

## 3 Results

### 3.1 Reported JE cases

**Figure 2:**
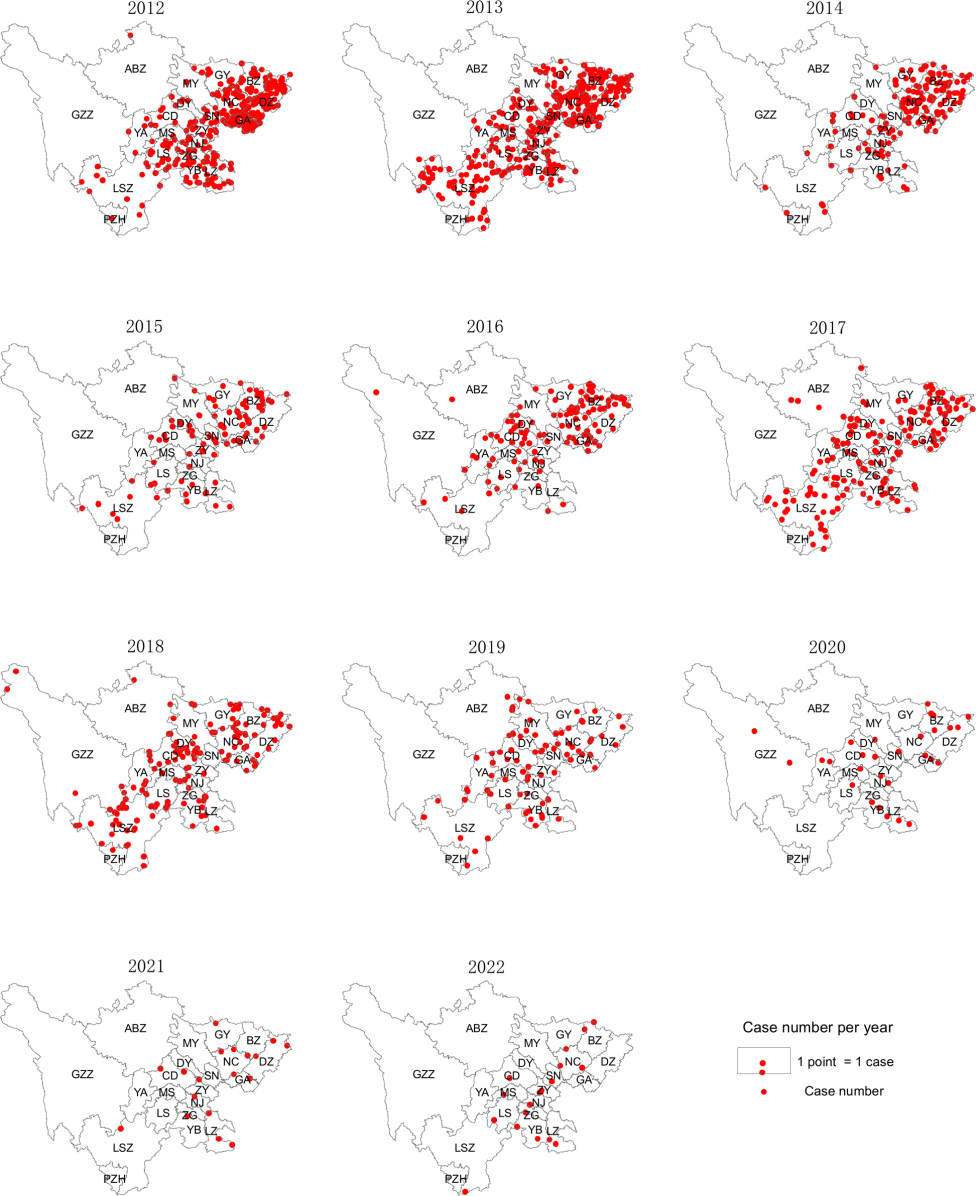
Spatial and temporal distribution of JE cases in the Sichuan province of China (2012–2022).

From 2012 to 2022, 1558 cases of JE were reported in the Sichuan province. The annual incidence of JE ranged between 0.0215/100,000 and 0.4557/100,000. The average annual incidence and mortality rates were 0.1724/100,000 and 0.0053/100,000, respectively. While the JE incidence and mortality rates in 2012 were 0.4075/100,000 and 0.0149/100,000, respectively, they decreased to 0.0215/100,000 and 0/100,000, respectively, in 2022, and there were no deaths recorded from 2021 onward (**Table 1**). The regional distribution of the reported cases of JE in the Sichuan province from 2012 to 2022 is shown in **Figure 2**.

**Table 1:** Annual incidence and mortality of JE in the Sichuan province of China (2012–2022).

Year	Case NO.	Incidence(1/10,0000)	Death NO.	Mortality (1/10,0000)
2012	332	0.4075	24	0.0149
2013	374	0.4557	28	0.0173
2014	159	0.1937	10	0.0062
2015	99	0.1216	4	0.0025
2016	118	0.1438	6	0.0037
2017	180	0.2179	8	0.0048
2018	145	0.1759	6	0.0036
2019	86	0.1031	4	0.0024
2020	29	0.0346	4	0.0024
2021	18	0.0215	0	0
2022	18	0.0215	0	0
Total	1558	0.1724	94	0.0053

### 3.2 Laboratory-confirmed JE cases

**Figure 3:**
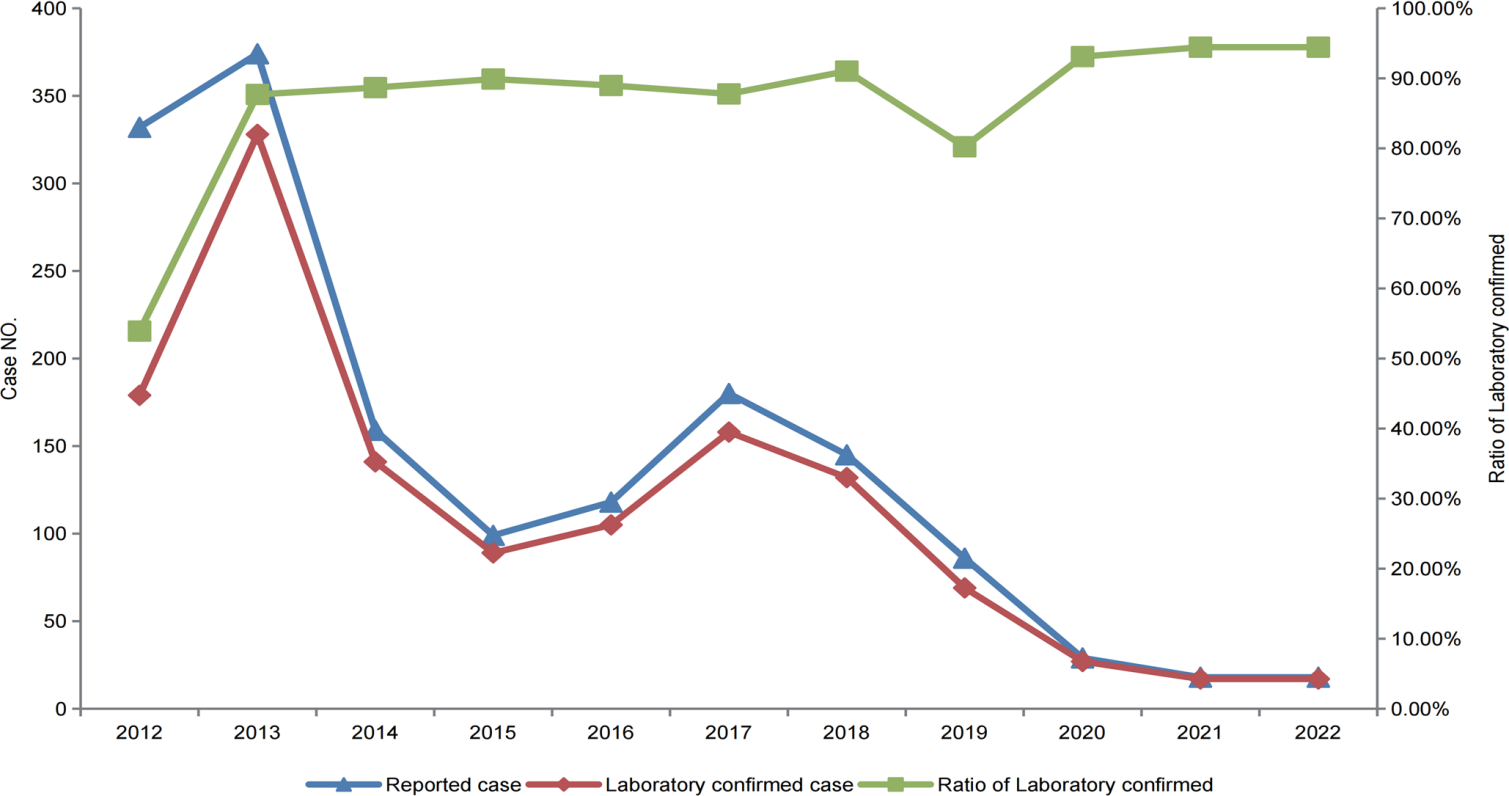
Reported and laboratory-confirmed cases of JE in the Sichuan province of China, from 2012–2022.

JEV-specific IgM was detected in the acute-phase serum of 1262 of the 1588 reported cases of JE in the Sichuan province from 2012 to 2022, with a positivity rate of 81% (1262/1588), while the other 296 patients tested negative for JEV using ELISA. CSF was collected from 218 of the 296 patients who were negative for JEV, and RT-PCR was performed to detect JEV genes but no JEV-specific genes were detected. Thus, there were 1262 laboratory-confirmed cases of JE among the 1558 reported cases of JE. The laboratory confirmation rate of the reported cases of JE in the Sichuan province has steadily increased yearly from 53.92% in 2012. In 2013, it increased to 87.7%, and in 2020, the laboratory confirmation rate for JE cases remained stable at >93% (**Figure 3**). Therefore, the gap between clinically and laboratory-confirmed cases of JE has been shrinking over time.

**Figure 4:**
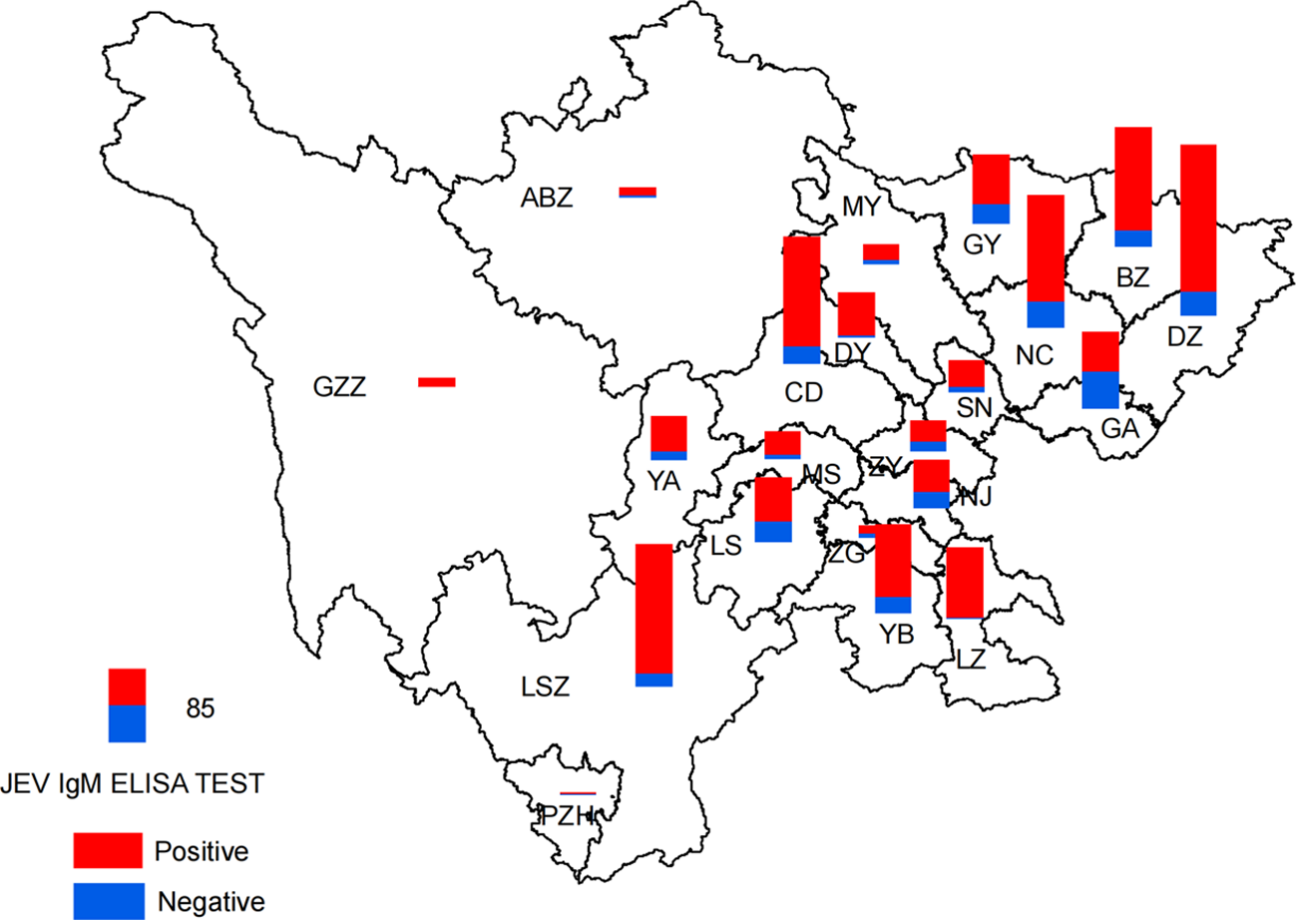
Spatial distribution of detection results of JEV IgM by ELISA in the Sichuan province of China (2012–2022).

Analysis of the geographic distribution of IgM testing for JEV revealed different rates of IgM positivity among the reported cases of JE in the 21 cities (prefectures). The laboratory confirmation rates for JE cases varied from 33.33% to 98.78%, highlighting a notable disparity in the rates of laboratory diagnoses across different regions (**Figure 4**).

#### 3.2.1 Gender

Among the 1262 laboratory-confirmed cases of JE detected between 2012 and 2022, 739 were male and 523 were female, reflecting a male-to-female ratio of 1.41:1 (**Table 2**).

**Table 2:** Epidemiological features of laboratory confirmed cases of JE in the Sichuan province, China (2012–2022).

Age-group	No. of cases													Peak Months	Ratio(Male : Female)
	2012	2013	2014	2015	2016	2017	2018	2019	2020	2021	2022	Total	Average Annual Incidence(1/100000)		2012-2022
0-15	176	303	126	79	83	107	66	35	15	16	11	1017	0.1122	Jul-Sep	1.38:1
16-40	2	8	10	6	11	27	41	16	3	1	5	130	0.0143	Jul-Sep	1.24:1
>40	1	17	5	4	11	24	25	18	9	0	1	115	0.0127	Jul-Sep	2.03:1
Total	179	328	141	89	105	158	132	69	27	17	17	1262	0.1392	Jul-Sep	1.41:1
>16	3	25	15	10	22	51	66	34	12	1	6	245	0.0270	Jul-Sep	1.55:1

#### 3.2.2 Seasonal pattern

**Figure 5:**
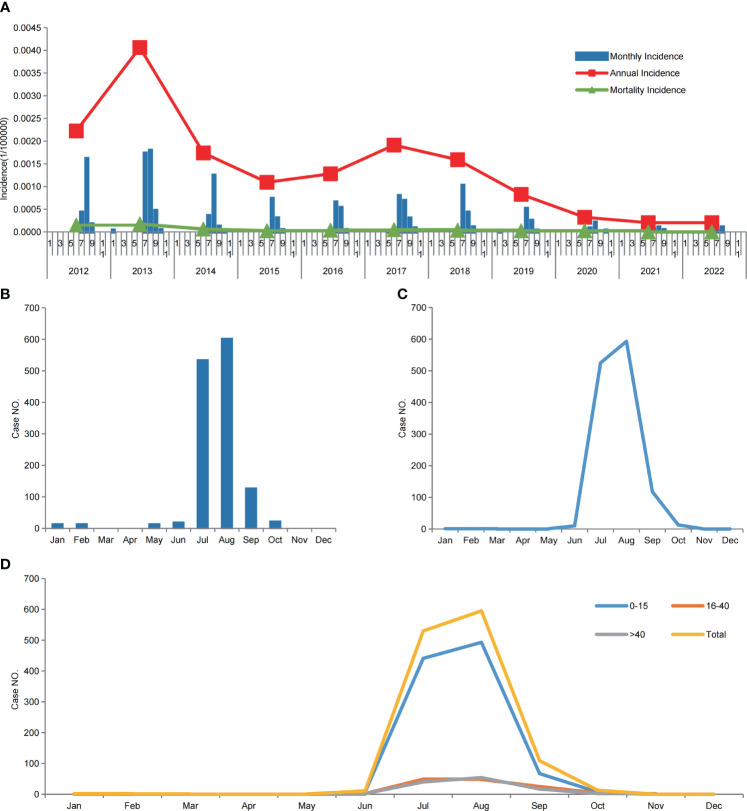
Japanese encephalitis in the Sichuan province of China (2012–2022). **(A)** Annual incidence and mortality, and monthly incidence of JE, from 2012 to 2022. **(B)** Monthly cumulative JE cases from 2012 to 2022. **(C)** The seasonal pattern of laboratory-confirmed cases of JE from 2012 to 2022. **(D)** The seasonal pattern of laboratory-confirmed cases of JE from 2012 to 2022, among the three age groups.

The distribution of JE cases showed a clear seasonal pattern. A total of 98% (1236/1262) of the JE cases occurred (**Figures 5A, B**) and peaked (**Figure 5C**) between July and September, in the Sichuan province.

#### 3.2.3 Geographical distribution

Between 2012 and 2022, laboratory-confirmed cases of JE were reported in all 21 cities (prefectures) in the Sichuan province. Most cases were concentrated in the eastern and southern regions of the Sichuan province, which have a warm and humid climate with mild winters, hot summers, and abundant precipitation. In contrast, the northwestern parts of the Sichuan province, characterized by a high altitude and frigid climate, had comparatively fewer cases (**Figures 2**, **4**).

#### 3.2.4 Epidemiological features of JE cases among different age groups

A statistical analysis of confirmed JE cases among different age groups was conducted, and the results showed that those who were 0–15 y old exhibited the highest incidence of JE in the Sichuan province, with an incidence rate of 0.1122/100,000 and male-to-female ratio of 1.38:1. Among those aged ≥16 y, the incidence rate was lower than that among those aged 0–15 y. JE cases peaked from July to September in all age groups (**Table 2**, **Figure 5D**).

Between 2012 and 2022, although the majority of confirmed cases of JE were in the 0–15-year-old group, there was an increase in adult cases (>16 y old) over the years. The number of adult cases increased from 3 in 2012 to 66 in 2018 (**Table 2**).

**Figure 6:**
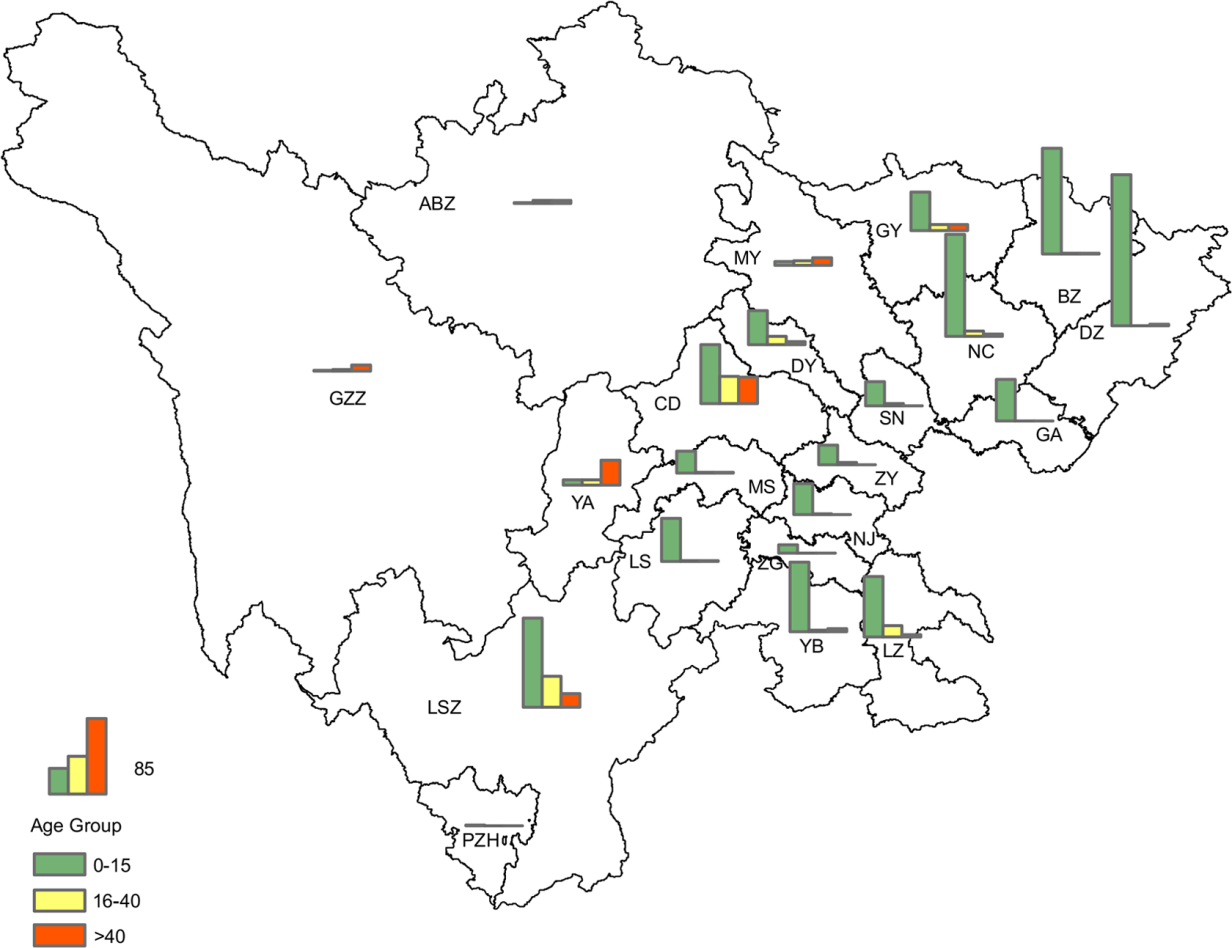
Geographical distribution of laboratory-confirmed cases of JE in the three age groups in the Sichuan province of China (2012–2022).

Geographic analysis was conducted on confirmed cases of JE in different age groups. The results showed that in the southeastern part of the Sichuan province, where the incidence rate of JE is relatively high, there was a higher proportion of cases in younger age groups. Conversely, in the adjacent central and western regions of the Sichuan province, where the incidence rate was relatively low, there was a higher proportion of cases in the older age groups, particularly among those older than 40 y of age (**Figure 6**).

### 3.3 Non-JE cases with confirmed pathogens

Of the 218 CSF samples collected from 296 serologically confirmed non-JE cases, RT-PCR was performed to detect JEV and 9 other viruses; none of the cases tested positive for JEV by RT-PCR. While EV, VZV, EBV, MuV, HHV-6, and HSV were detected in 62 cases, there were 5 cases of involving multiple infections. MV, RUV, and CMV were not detected in any of the cases.

The results indicated that of the 296 serologically confirmed non-JE cases, 62 cases were positive for EV and other 6 viruses. The infection rate was 21% (62/296), and these 62 cases were classified as non-JE cases with confirmed pathogens; the majority of the 62 cases involved single infections with EBV, EV, or HSV-1. Some patients exhibited mixed infections (**Table 3**).

**Table 3:** Epidemiological features of non-JE cases with confirmed pathogens in the Sichuan province of China (2012–2022).

**Pathogen**	**Ratio(Male : Female)**		**Age Group**			**Case number per Year**										**Co-infection** **Yes**	**Co-infection No**
	**Male**	**Female**	**<15**	**16-40**	**>40**	**2012**	**2013**	**2014**	**2016**	**2017**	**2018**	**2019**	**2021**	**2022**	**Total**		
EV	10	7	15	2	0	0	3	4	0	0	3	7	0	0	17		√
EBV	9	8	11	1	5	3	3	1	1	4	1	3	0	1	17		√
HSV-1	5	8	6	1	6	0	1	3	2	0	0	6	1	0	13		√
HSV-2	1	0	0	1	0	0	1	0	0	0	0	0	0	0	1		√
VZV	4	2	3	0	3	0	0	0	1	0	5	0	0	0	6		√
MuV	2	0	2	0	0	0	1	0	0	0	1	0	0	0	2		√
HHV-6	1	0	1	0	0	0	0	1	0	0	0	0	0	0	1		√
HSV-1/2	0	1	1	0	0	0	1	0	0	0	0	0	0	0	1	√	
EB/HSV-1	0	1	1	0	0	0	0	0	0	1	0	0	0	0	1	√	
EV/HHV-6	0	1	1	0	0	0	0	0	0	0	0	1	0	0	1	√	
EV/EB	2	0	2	0	0	1	0	0	0	0	1	0	0	0	2	√	
Total	34	28	43	5	14	4	10	9	4	5	11	17	1	1	62	/	/
Ratio	1.21:1		69.35%	8.06%	22.58%	6.45%	16.13%	14.52%	6.45%	8.06%	17.74%	27.42%	1.61%	1.61%	/	/	/

In terms of the temporal distribution of the non-JE cases with confirmed pathogens, EBV infection cases had the widest temporal distribution, with only 3 y not detected within the 11-year period. EV infections were detected mainly in the years 2013–2014 and 2018–2019, for a total of 4 y. HSV-1 was detected over five years, with the highest number of cases detected in 2019 (six cases). The temporal distribution of VZV, MuV, HSV-2, and HHV-6 infection cases was the shortest, at only–1–2 y (**Table 3**).

**Figure 7:**
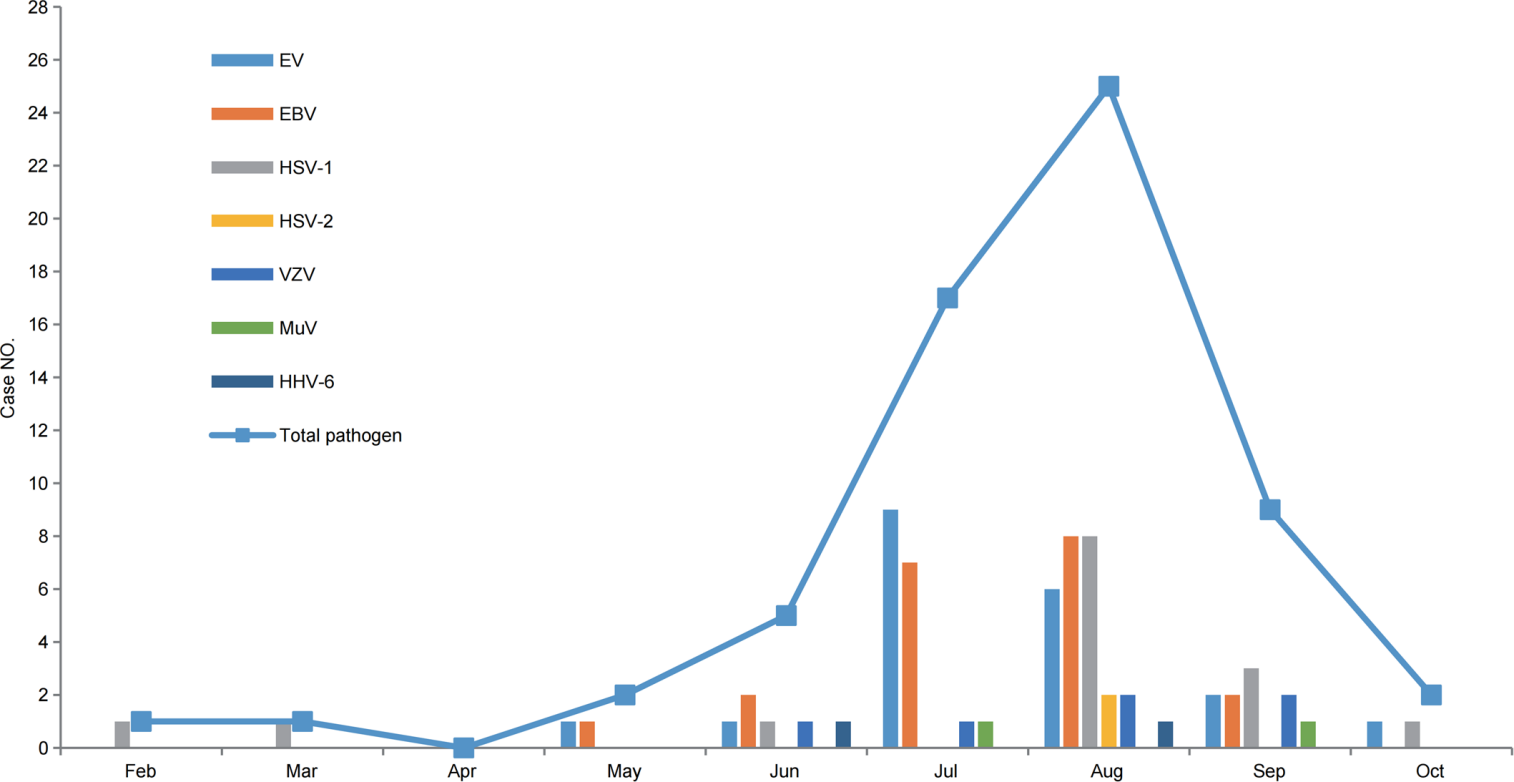
The cumulative number of non-JE cases with confirmed pathogens in the Sichuan province of China, monthly (2012–2022).

**Figure 8:**
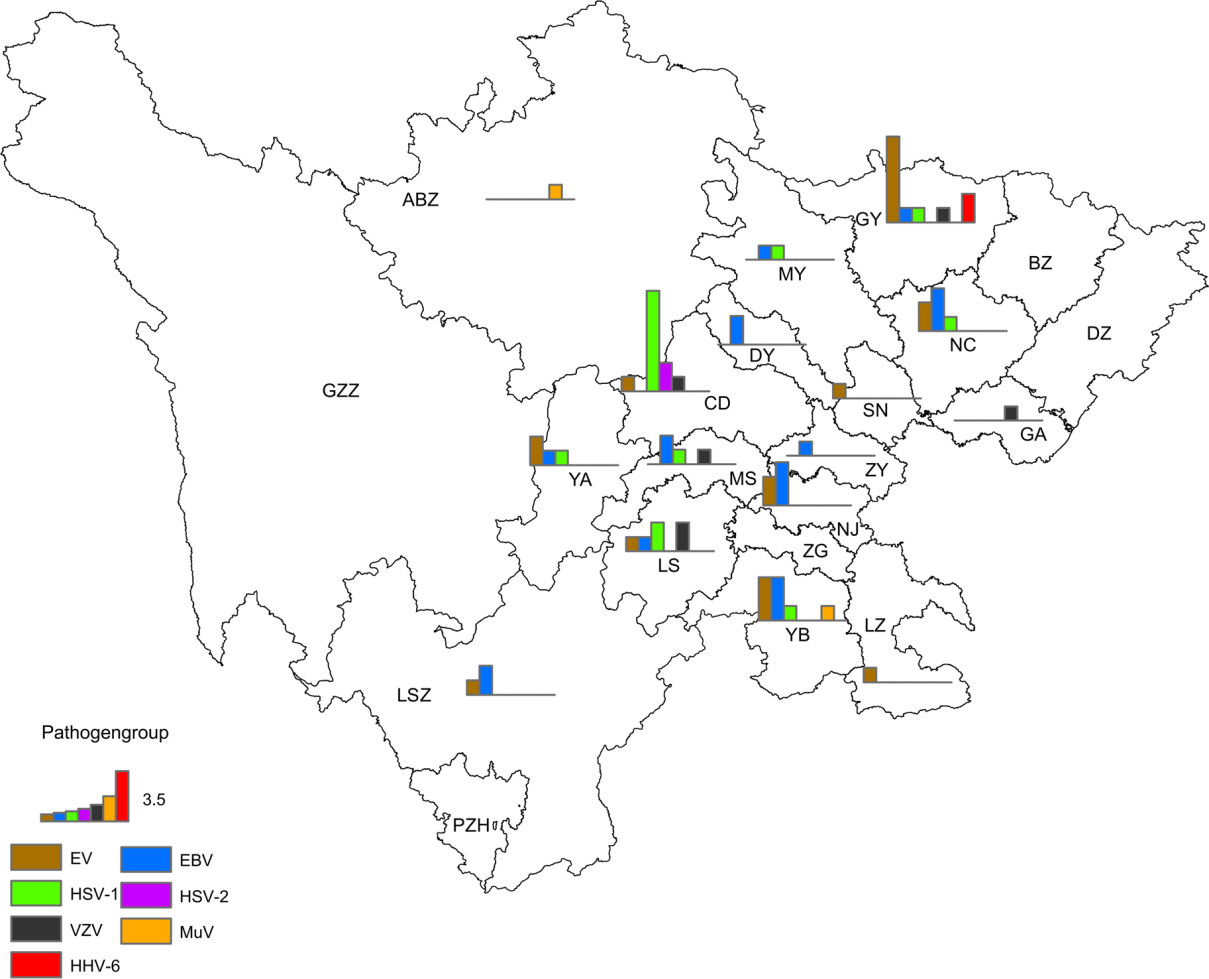
Geographical distribution of non-JE cases with confirmed pathogens in the Sichuan province of China (2012–2022).

Among the 62 non-JE cases with confirmed pathogens, 34 were male and 28 were female, reflecting a male-to-female ratio of 1.21:1; there were slightly more males than females. Regarding age groups, 69.35% (43/62) of the cases belonged to the 0–15 y group, 22.58% (14/62) of the cases belonged to the >40 y group, and 8.06% (5/62) of the cases belonged to the 16–40 y group (**Table 3**). Regarding the seasonal pattern, cases were distributed from February to October, with a total of 82.26% (51/62) cases occurring between July and September (**Figure 7**). From the geographical distribution, of 21 cities (prefectures), 17 (17/21) had cases positive for viruses other than JEV (**Figure 8**).

### 3.4 Unknown viral encephalitis cases among the reported cases

Attempts to detect the viruses in the 1558 reported JE cases in the Sichuan province from 2012 to 2022 revealed 1262 laboratory-confirmed JE cases, 62 non-JE cases with confirmed pathogens, and 234 cases classified as unknown viral encephalitis. Among the 234 cases of unknown viral encephalitis, 130 were male and 104 were female, reflecting a male-to-female ratio of 1.25:1; 94.87% (222/234) of the cases involved those aged 0–15 y, while there were only 3 cases in the 16–40 y age group and 9 cases in the >40 y age group, accounting for a cumulative 5.13% of cases; the peak incidence occurred between July and September (**Table 4**).

**Table 4:** Epidemiological features of unknown viral encephalitis in the Sichuan province of China (2012–2022).

	Ratio(Male : Female)		Age Group			Case number of Month					
	Male	Female	<15	16-40	>40	May	Jun	Jul	Aug	Sep	Total
Case NO.	130	104	222	3	9	4	11	56	141	22	234
Ratio	1.25:1		94.87%	1.28%	3.85%	1.71%	4.70%	23.93%	60.26%	9.40%	/

## 4 Discussion

In this study, acute-phase serum and CSF samples of reported cases of JE (including some clinically diagnosed cases) in the Sichuan province from 2012 to 2022 were analyzed by serological and genetic testing. The results showed that, apart from JE cases, 19% (296/1588) were non-JE cases, including non-JE cases with confirmed pathogens and unknown viral encephalitis cases.

With JE being highly endemic in China, the gradual promotion of JE vaccination has reduced the incidence rate from 20.92/100,000 in1971 to 0.12/100,000 in 2011 (Gao et al., 2014; Li et al., 2014). In particular, since the Chinese government included the JE vaccine in the Expanded Program on Immunization in 2008, those aged between 0–15 years can receive free JE vaccines, and consequently, JE incidence in China has decreased significantly (Gao et al., 2014; Li et al., 2014; Li et al., 2016). In 2021 and 2022, there were 209 and 153 reported JE cases nationwide, with 3 and 6 deaths, respectively (Division of Infectious Disease, Chinese Center for Disease Control and Prevention, 2022). The public health burden of JE in China is decreasing annually, and the incidence of JE among children has been greatly reduced by the extensive promotion of vaccinations; however, the incidence of JE among adults has increased annually. There were several outbreaks of adult JE in 2006, 2009, and 2013 (Hu et al., 2017; Li, X. et al., 2019; Wu et al., 2021). The Ningxia province had reported 162 JE cases in 2018, with 31 deaths. Among the 162 JE cases, 146 cases belonged to the >40 y age group, and only two cases belonged to the 0–15 y age group, indicating that 90% (146/162) of the JE cases in Ningxia in 2018 were adult cases (Liu et al., 2020). The analysis of laboratory-confirmed cases of JE in various age groups in the Sichuan province from 2012 to 2022 showed that among the1262 laboratory-confirmed cases of JE,1017 cases involved children (0–15 y old) and 245 cases involved adults (>16 y old). Although the incidence rate of cases in adults was much lower than that in children, the number of JE cases among children decreased from 303 in 2013 to 11 in 2022 (**Table 2**). In contrast, JE cases in adults showed a consistent increase from 3 cases in 2012 to 66 cases in 2018. The clinical analysis of hospitalized patients with laboratory-confirmed cases of JE in the Sichuan province by the West China Hospital of Sichuan University from January 2011 to September 2018 showed that patients who were ≥50 y of age had more complex clinical symptoms, more severe manifestations, and higher mortality rates (Xiong et al., 2019). The increase in JE cases among adults in the Sichuan province may be a result of multiple reasons. First, adults have not been vaccinated with the JE vaccine in their childhood, resulting in a lack of immunity against JEV infection, and second, viruses causing encephalitis are not only diverse but also lack specific clinical manifestations. Therefore, it is difficult to distinguish the types of viral infections without virus-specific detection techniques in the laboratory. In addition to the 1262 laboratory-confirmed cases of JE, this study also identified 62 non-JE cases with EV and 6 other viral infections (**Table 3**), and 234 unknown viral encephalitis cases (**Table 4**). Adults infected with JEV can be infected by different genotypes. It is well known that JEV is divided into five genotypes (GI-V) (Pan et al., 2019), and the current JE vaccines used worldwide, whether JE-inactivated (P3) or JE-attenuated live (SA-14-14-2 strain) vaccines, are all prepared from the genotype III JEV (World Health Organization, 2014). In 2015, a patient in South Korea was infected with JEV after full JE vaccination, and genetic analysis of the virus isolated from the patient’s CSF showed that the patient was infected with the genotype V JEV (Woo et al., 2020). Previous studies have indicated that vaccines prepared from the genotype III JEV have low protective efficacy against infection by the genotype V JEV (Cao et al., 2016).

More than 100 types of viruses have been found to cause central nervous system infections, and new viruses causing encephalitis, such as the Nipah virus, are constantly emerging. Research shows that the global annual incidence of viral central nervous system infections ranges from 3.5/100000 to 7.4/100000 (Swanson and McGavern, 2015). Although viral encephalitis can be caused by several viruses, such as JEV, VZV, or EV, the initial clinical symptoms of viral encephalitis are similar, including fever, headache, convulsions, and disruption of consciousness. In this study, 62 non-JE cases with confirmed pathogens (**Table 3**) and 234 unknown viral encephalitis cases (**Table 4**) were detected among the clinical specimens diagnosed as JE. This not only suggests that a large number of misdiagnosed cases may exist among the cases diagnosed with JE based on clinical symptoms, but also indicates that there are a large number of sporadic viral encephalitis cases in the local population in addition to JEV infection, such as EV, EBV, and HSV infections, as well as cases of mixed infections involving multiple viruses, such as EV and HSV infections, which further emphasizes the importance of laboratory detection for specimens from patients with viral encephalitis.

In 2006, 1837 JE cases were reported in the Guizhou province, which is located in southwest China, similar to the Sichuan province, and laboratory detection of acute-phase serum or CSF collected from 1382 patients showed that 87.5% (1,210/1,382) of the cases were laboratory-confirmed JE cases. The specimens of the other 172 non-JE patients were analyzed for IgM of various viruses. The acute-phase serum analysis results showed that 58.2% (39/67) of the cases were positive for MuV IgM, 11.9% (8/67) of the cases were positive for ECHO IgM, 9.0% (6/67) of the cases were positive for VZV IgM, 3% (2/67) of the cases were positive for COXV IgM, and 1.5% (1/67) of the cases were positive for CMV IgM. EBV, VZV, or MV were not detected in the patient specimens (Ye et al., 2010). From 2006 to 2008, active surveillance studies on acute meningitis and encephalitis syndrome were conducted in four counties in China (the Hebei, Henan, Shandong, and Guangxi provinces). The results of the detection of 4513 cases in the acute meningitis and encephalitis syndrome surveillance program showed that 9.2% of the cases were laboratory-confirmed cases of JE, and EV-specific IgM and VZV-specific IgM were detected as positive as well (Yin et al., 2010). From January to December 2010, serum and CSF specimens were collected from 189 patients with viral encephalitis at Mangshi and Ruili hospitals in the Yunnan province, within 6 days of hospitalization. Among the specimens, only 22 cases were positive for JEV-specific IgM; additionally, 5 cases were positive for MuV-specific IgM, 8 cases were positive for ECHO specific IgM, and 5 cases were positive for COXV-specific IgM. The remaining cases were classified as unknown viral encephalitis cases (Feng et al., 2013). These results indicate that there are many cases of respiratory viral and EV infections in Chinese children with viral encephalitis, apart from JEV infection.

In this study, a significant number of non-JEV cases were detected through the laboratory analysis of acute-phase specimens from reported cases of JE in the Sichuan province from 2012 to 2022. This indicates that there were non-laboratory-confirmed cases among the reported cases of JE in the past 11 years, which greatly affected the accuracy of information regarding locally reported cases of JE. Therefore, it is necessary to strengthen the laboratory detection of JE to ensure that each reported case of JE is confirmed through laboratory testing, and avoid cases with a mixture of other viral infections. Additionally, clinicians should be promptly informed about the test result if there are viruses other than JEV, such as EV, EBV, and other encephalitis viruses, so they can adjust the treatment strategy in a timely manner to reduce the uncertainty in treatment success. This study analyzed the relationship between reported cases and laboratory-confirmed cases of JE. These research results are not only important for the Sichuan province but also provide a valuable reference for laboratory detection of various infections in China and regions outside China.

## Data availability statement

The original contributions presented in the study are included in the article/Supplementary Material. Further inquiries can be directed to the corresponding author.

## Ethics statement

The studies involving humans were approved by Ethics Committee of Sichuan CDC. The studies were conducted in accordance with the local legislation and institutional requirements. The human samples used in this study were acquired from primarily isolated as part of your previous study for which ethical approval was obtained. Written informed consent for participation was not required from the participants or the participants’ legal guardians/next of kin in accordance with the national legislation and institutional requirements. Written informed consent was obtained from the individual(s), and minor(s)’ legal guardian/next of kin, for the publication of any potentially identifiable images or data included in this article.

## Author contributions

WL: Methodology, Project administration, Resources, Writing – original draft, Writing – review & editing. YF: Formal analysis, Software, Visualization, Writing – original draft. HZ: Data curation, Investigation, Writing – original draft. MJ: Data curation, Investigation, Writing – original draft. JZ: Methodology, Supervision, Writing – review & editing. SL: Methodology, Supervision, Writing – original draft. NC: Data curation, Writing – original draft. SH: Supervision, Writing – review & editing. KZ: Data curation, Writing – original draft. SF: Methodology, Writing – original draft. HW: Methodology, Writing – review & editing. GL: Conceptualization, Supervision, Validation, Writing – review & editing.
